# Spinal ependymoma in a patient with Kabuki syndrome: a case report

**DOI:** 10.1186/s12881-015-0228-4

**Published:** 2015-09-05

**Authors:** Davide Roma, Paolo Palma, Rossella Capolino, Lorenzo Figà-Talamanca, Francesca Diomedi-Camassei, Francesca Romana Lepri, Maria Cristina Digilio, Carlo Efisio Marras, Raffaella Messina, Andrea Carai, Franco Randi, Angela Mastronuzzi

**Affiliations:** University Department of Pediatrics, Bambino Gesù Children’s Hospital, IRCCS - “Tor Vergata” University, Rome, Italy; Department of Neuroscience and Neurorehabilitation, Neurosurgery Unit, Bambino Gesù Children’s Hospital, IRCCS, Rome, Italy; Medical Genetics Unit, Bambino Gesù Children’s Hospital, IRCCS, Rome, Italy; Department of Radiology, Unit of Neuroradiology, Bambino Gesù Children’s Hospital, IRCCS, Rome, Italy; Department of Anatomical Pathology, Bambino Gesù Children’s Hospital, IRCCS, Rome, Italy; Cytogenetics and Molecular Genetics Unit, Bambino Gesù Children’s Hospital, IRCCS, Rome, Italy; Department of Hematology/Oncology and Stem Cell Transplantation, Bambino Gesù Children’s Hospital, IRCCS, Rome, Italy

**Keywords:** Kabuki syndrome, Spinal ependymoma, KMT2D mutation, Cancer predisposition syndromes

## Abstract

**Background:**

Kabuki syndrome is a rare disorder characterized by the association of mental retardation and postnatal growth deficiency with distinctive facial appearance, skeletal anomalies, cardiac and renal malformation. Two causative genes have been identified in patients with Kabuki syndrome. Mutation of *KMT2D* (*MLL2*) was identified in 55–80 % of patients, while 9–14 % of *KMT2D* negative patients have mutation in KDM6A gene. So far, few tumors have been reported in patients with Kabuki syndrome. We describe the first case of a patient with spinal ependymoma and Kabuki syndrome.

**Case presentation:**

A 23 years old girl followed at our Center for *KMT2D* mutated Kabuki syndrome since she was 4 years old presented with acute lumbar pain and intermittent tactile hyposthenia of the feet. Spine magnetic resonance revealed a lumbar endocanalar mass. She underwent surgical resection of the lesion and histologic examination showed a tanycytic ependymoma (WHO grade II).

**Conclusion:**

Kabuki syndrome is not considered a cancer predisposition syndrome. Nonetheless, a number of tumors have been reported in patients with Kabuki syndrome. Spinal ependymoma is a rare disease in the pediatric and young adult population. Whereas NF2 mutations are frequently associated to ependymoma such an association has never been described in Kabuki syndrome. To our knowledge this is the first case of ependymoma in a *KMT2D* mutated Kabuki syndrome patient. Despite *KMT2D* role in cancer has previously been described, no genetic data are available for previously reported Kabuki syndrome patients with tumors. Nonetheless, the association of two rare diseases raises the suspicion for a common determinant.

## Background

Kabuki syndrome (KS), known as Kabuki make-up syndrome or Niikawa–Kuroki syndrome, is a rare disorder (1:32000 live births) [1] firstly described by Kuroki et al. in 1981 in patients that had a distinctive facial appearance, skeletal anomalies, cardiac and renal malformations, mild to moderate mental retardation and postnatal growth deficiency [2]. KS is associated with numerous alteration in body system and apparata such as neurological abnormalities, impairment in growth, endocrinological findings, cardiac and otolaryngological malformations, and other clinical manifestations [3]. Two causative genes have been identified in KS patients. In particular, mutation in *KMT2D* at 12q13.12 account for 55–80 % of the patient [4], whereas 9–14 % of *KMT2D* negative patients have deletions or mutation in KDM6A gene at Xp11.3 [5]. The absence of genetic mutations is not an exclusion criterion for clinical diagnosis of KS.

Some cases in literature reported the association with KS and cancer even if there are no conclusive findings of the increased risk for cancer in patients with the syndrome and there are no data about the real incidence of cancer in KS [3]. Notably, only scattered case reports are found in the literature of KS associated with pre-B-ALL, hepatoblastoma, neuroblastoma, Burkitt lymphoma and synovial sarcoma [6–11].

Ependymoma is a tumor derived from the ependymal cells lining the ventricular system and is the third most common central nervous system (CNS) tumor in childhood. Most cases are located intracranially, in particular in the posterior fossa; spinal location is less frequent [12]. Ependymomas are classified as subependymomas and myxopapillary (grade I), classic (grade II), and anaplastic (grade III), according to the World Health Organization (WHO). In the pediatric population, half of the cases are diagnosed in children of less than 5 years of age [13]. Most cases of ependymoma involve adult patients, in particular the highest rates were observed in the 45–64 years age group. On the contrary, ependymomas are five times more malignant in the age group < 19 years than in adults where the incidence rate ratio (IRR) of malignant to borderline malignant tumor is about 1.5. Spinal cord/cauda equina is the primary site in 52.1 % of all cases in adults, but in children (age < 19 years) it is involved in only about 20 % of cases [14].

We describe a KS girl with the diagnosis of grade II ependymoma of the filum terminale.

## Case presentation

A girl born at the 40th week of gestation with a weight of 2,380 g presented hypotonia and submucous cleft palate, surgically corrected when she was 6 years old. Physical examination at 3 years of age showed facial anomalies including arched eyebrows with lateral thinning, long palpebral fissures with lateral eversion of the lower eyelid, long eyelashes, large prominent ears with dysplastic helices, and a depressed nasal tip, suggestive for KS. She also had hypotonia, joint laxity, retarded motor developmental milestones and mild cognitive deficit. Two-dimensional color-doppler echocardiography and renal ultrasound examination were normal. Genetic testing of *KMT2D* gene, performed by target resequencing on the MiSeq (Illumina) platform showed the heterozygosis deletion of two bases c.16085_16086delAG; the identified variation resulted at protein level in the nonsense mutation p.Lys5362Serfs*96.

**Fig. 1:**
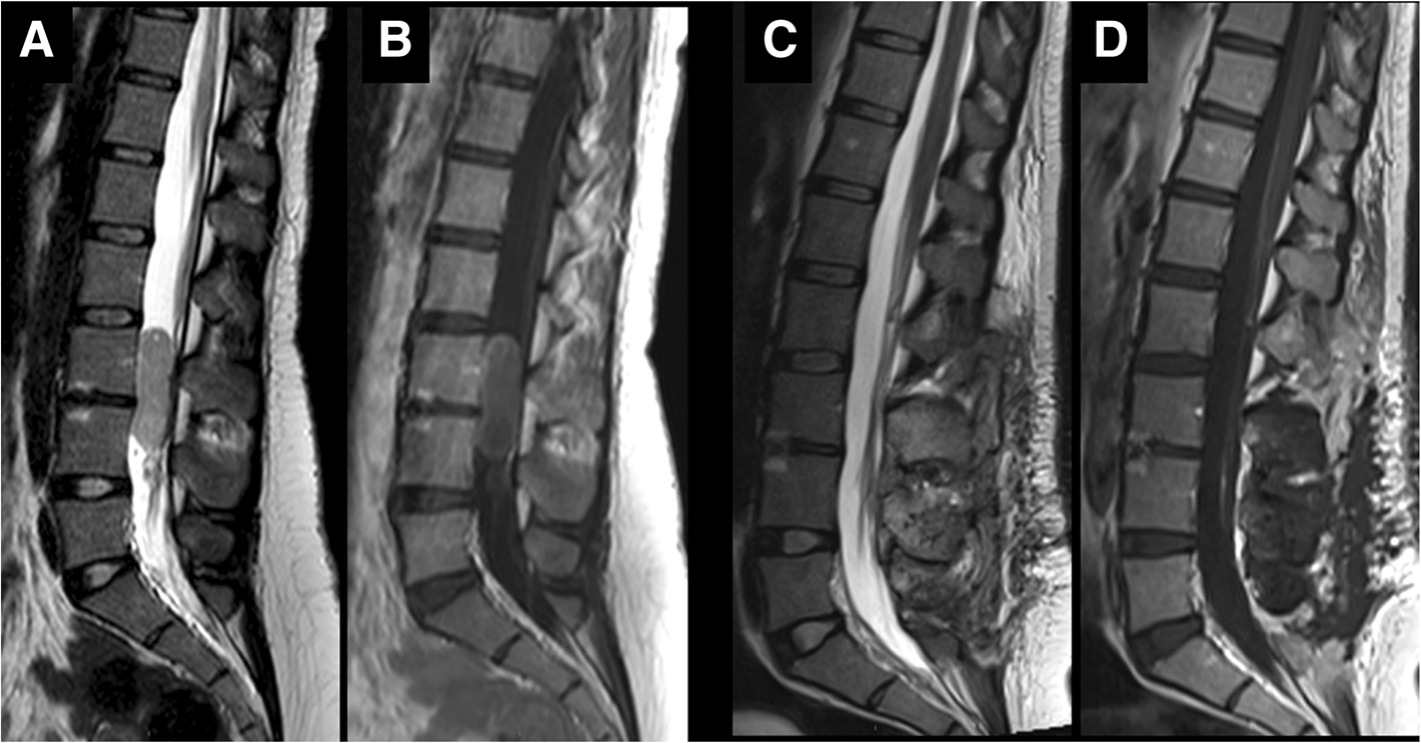
Spine MRI, sagittal pre-operative and post-operative images. Sagittal pre-operative TSE T2 WI (**a**) and T1 WI with Gd (**b**) show a lumbar endocanalar mass extending from L3 to L4, isointense on T1 and T2 WI with peripheral contrast enhancement. Sagittal post-operative TSE T2 WI (**c**) and T1 WI with Gd (**d**) show total resection of the tumor

**Fig. 2:**
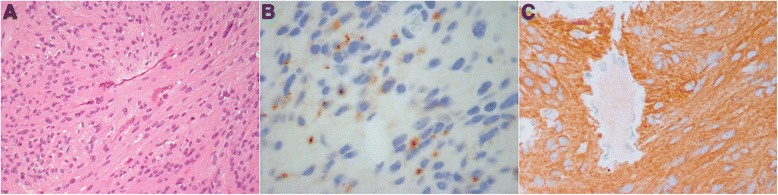
Photomicrographs of ependymoma of the filum terminale. A homogeneous proliferation of oval/elongated cells embedded in eosinophilic fibrillary stroma; a fascicular distribution and a vaguely perivascular pattern of growth are observed (**a**). At immunohistochemistry, a focal dot-like positivity for epithelial membrane antigen (EMA) is evident (**b**). Diffuse positivity of neoplastic cells for glial fibrillary acidic protein (GFAP +++) (**c**)

At the age of 23, she presented with intermittent tactile hypoesthesia of the feet and worsening lumbar pain. Magnetic resonance imaging (MRI) of the spine revealed the presence of a lumbar endocanalar mass extending from L3 to L4, isointense on T1 and T2 weighted images with peripheral contrast enhancement. The lesion had a maximum cranial-caudal diameter of 45 mm with diffuse compression and posterior displacement of spinal nerve roots. At surgery, an L3 to L5 laminotomy was performed and gross total resection of a clivable tumor arising from the filum terminale accomplished (Fig. 1). No neurological complications occurred. Histology revealed a monomorphic proliferation of elongated cells with mild nuclear atypia, surrounded by eosinophilic fibrillary stroma with a fascicular or vaguely perivascular pattern of growth. Cells showed diffuse positivity for glial fibrillary acidic protein (GFAP +++) and dot-like positivity for epithelial membrane antigen (EMA). The mitotic index assessed by immunohistochemical staining against anti-Ki67 was about 3–5 % (Fig. 2). These findings led to the diagnosis of ependymoma, likely the tanycytic type (WHO grade II). On the basis of site of the lesion, extent of resection, histology and age of the patient, no other treatment was offered after surgical resection. She remains disease free fourteen months after diagnosis.

## Conclusion

KS is a genetic syndrome firstly described in 1981. Patients present with mental and postnatal growth retardation associated with a characteristic facial appearance that resemble that of Kabuki Japanese theatre. Since KS is such a rare disease, no data are found in the literature about the incidence of cancer and the overall survival of patients with the syndrome. First cases described in the literature are now forty years old. These observations confirm that patients can survive to adulthood. The heterogeneity of anatomical and functional defects associated with KS can contribute in different ways to the long-term prognosis [15]. The genetic basis of KS were identified in 2010 and firstly associated to the mutation in *KMT2D* located in the chromosome 12 [4]. *KMT2D* is part of the lysine methyltransferase superfamily that are a group of evolutionarily conserved transcriptional regulators. The role of *KMT2D* is the methylation of histone 3 lysine 4 that acts as an activation mark [16, 17]. Guo et al. reported that the *KMT2D* deficiency modifies cancer cell proliferation and cell migration. Analysis of histone H3 modifications revealed that *KMT2D* is essential for maintaining the level of global H3K4 monomethylation and that its enzymatic SET domain is directly responsible for this function. Because they found that a majority of *KMT2D* binding sites were located in regions of potential enhancer elements, they supposed that these findings revealed the possible role of *KMT2D* in tumorigenesis [16]. Kabuki syndrome mutations C1430R and C1471Y reduce histone binding and catalytic activity of *KMT2D* [18]. The same reduction of function of *KMT2D*, due to somatic truncating mutations, has been reported in association to childhood cases of medulloblastoma and non-Hodgkin lymphoma [16].

Up to now, there are no available data regarding *KMT2D* mutation status in patients with KS that develop cancer. One study in a large cohort of pediatric cancer patients showed that the incidence of monogenic syndromes was higher than expected in the general population and that diagnoses of these syndromes had frequently been missed. Considering that 1 patient out of 1073 had been diagnosed with KS [6], Bogershausen et al. reported that the hypothesis of a KS predisposition to malignancies remains questionable [3].

To our knowledge, this is the first case of spinal ependymoma described in KS. Spinal ependymoma is a rare tumor of adults that represents about 60 % of spinal cords tumors. Pathophysiology of this tumor is not known so far but there is a high incidence (33 %) of spinal low grade ependymoma in patients with type 2 neurofibromatosis [19].

In conclusion, despite KS is not considered to be a cancer predisposition syndrome, an increasing number of tumors is being reported in these patients. No previous genetic data are available from KS patients developing tumors. *KMT2D* mutation is now found in most KS cases and shows a role in cancerogenesis. The rarity and heterogeneity of tumors descripted in patients with KS doesn’t permit to indicate an active oncologic surveillance in this patients. However we think that the description of new cases and the genetic characterizations of these patients can help us to better know the role of KMT2D mutation and the natural history of KS.

## Consent

Written informed consent was obtained from the patients’ parents for publication of these Case report and any accompanying images. A copy of the written consent is available for review by the Editor of this journal.

## Abbreviations

KS: Kabuki syndrome
CNS: Central nervous system
WHO: World Health Organization
IRR: Incidence rate ratio
MRI: Magnetic resonance imaging
EMA: Epithelial membrane antigen
